# Usutu virus: A new threat?

**DOI:** 10.1017/S0950268819001213

**Published:** 2019-07-04

**Authors:** M. Clé, C. Beck, S. Salinas, S. Lecollinet, S. Gutierrez, P. Van de Perre, T. Baldet, V. Foulongne, Y. Simonin

**Affiliations:** 1 Pathogenesis and Control of Chronic Infections, University of Montpellier, Inserm, EFS, Montpellier, France; 2 UPE, Anses Animal Health Laboratory, UMR1161 Virology, INRA, Anses, ENVA, Maisons-Alfort, France; 3 ASTRE, CIRAD, INRA, University of Montpellier, Montpellier, France; 4 Pathogenesis and Control of Chronic Infections, University of Montpellier, Inserm, EFS, CHU Montpellier, Montpellier, France

**Keywords:** Arboviruses, virology (human) and epidemiology, virology

## Abstract

Usutu virus (USUV) is an emerging arbovirus that was first isolated in South Africa in 1959. This *Flavivirus* is maintained in the environment through a typical enzootic cycle involving mosquitoes and birds. USUV has spread to a large part of the European continent over the two decades mainly leading to substantial avian mortalities with a significant recrudescence of bird infections recorded throughout Europe within the few last years. USUV infection in humans is considered to be most often asymptomatic or to cause mild clinical signs. Nonetheless, a few cases of neurological complications such as encephalitis or meningoencephalitis have been reported. USUV and West Nile virus (WNV) share many features, like a close phylogenetic relatedness and a similar ecology, with co-circulation frequently observed in nature. However, USUV has been much less studied and in-depth comparisons of the biology of these viruses are yet rare. In this review, we discuss the main body of knowledge regarding USUV and compare it with the literature on WNV, addressing in particular virological and clinical aspects, and pointing data gaps.

## Usutu virus: a flavivirus of African origin

Among emerging viruses, Usutu virus (USUV) has recently attracted the attention of the scientific community due to its extensive spread in Europe. USUV is an arbovirus of the *Flaviviridae* family and of the *Flavivirus* genu*s*, comprising more than 70 members. Flaviviruses include some of the most pathogenic arboviruses for humans, such as West Nile virus (WNV), dengue virus, yellow fever virus, Zika virus as well as Japanese encephalitis virus (JEV) [1]. USUV is a member of the Japanese encephalitis serocomplex and is phylogenetically close to JEV and WNV [2, 3]. Its name derives from the Usutu River in Swaziland, in Southern Africa. USUV was first identified in 1959 by McIntosh as part of a study on the prevalence of viruses in arthropods in South Africa during which USUV was isolated from field-caught *Culex neavei* mosquitoes through intracerebral inoculation of newborn mice [4, 5]. Then, it was also isolated from the bird-biting mosquito *Mansonia aurites* in Uganda [4]. USUV is an enveloped virus of approximately 40–60 nm in diameter, with a single-stranded RNA of positive polarity comprised of 11 064 nucleotides harbouring a 5′ N7-methylguanosine-triphosphate cap but lacking a polyA tail at the 3′ end [6]. The genome of USUV comprises a single open reading frame coding for a polyprotein of 3434 amino acids that, after cleavage, generates to three structural proteins (capsid C, premembrane prM and envelope E) and eight non-structural proteins (NS1/NS1’, NS2a, NS2b, NS3, NS4a, 2K, NS4b and NS5) [2]. The capsid protein (C) forms the central body of the virion and is associated with the viral RNA. The prM protein is required for virion assembly and maturation of virions through the folding of the envelope glycoprotein (E) that participates in various aspects of the viral cycle such as attachment and fusion to the cell membrane [7]. The non-structural proteins (NS) of flaviviruses is associated with the endoplasmic reticulum to form replication complexes in which NS5 ensures viral RNA replication by its RNA-dependent RNA polymerase activity [6]. Similarly to other flaviviruses, viral replication takes place in the cytoplasm of infected cells. The NS5 protein, which is highly conserved among USUV strains, has a methyltransferase domain required for the addition of the cap element at the 5′ end of the viral genomic RNA [7]. Phylogenetic studies based on the nucleic acid sequence of the NS5 gene have shown that USUV strains isolated in different regions of the world can be divided into eight lineages: three African and five European [8], and that the level of genetic relatedness depends on their geographical origin and on the host from which they have been isolated. A comparative analysis of USUV genomes reveals specific amino acid mutations linked with the geographical origin of the isolate and the hosts involved. These mutations are found particularly in C (A120V), NS4B (M16I), prM (Y120N), as well as E (G195R) [9–11].

## Tropism and pathogenesis

USUV has been shown to infect a large number of cell lines or primary cells from different species (e.g. human (dendritic and Hela cells), equine (ED), bovine (MDBK), porcine (PK-15), rabbit (RK-13), canine (MDCK, DK), feline (CR), hamster (BHK-21, BF), rat (C6), turtle (TH1), birds (GEF), monkey (LLC-MK2, Vero cells)) [12, 13]. Cytopathic effects have been observed in Vero, GEF, CRFK, DN1.Tr, E, EA.hy.926, FoLu, OHH1.K, OK, PK(15), Sf 1 Ep, A549, Hep-2, KB and Mv 1 Lu cell lines [12, 14, 15]. USUV, like for other flaviviruses, can also infect murine mature neurons and microglial cells *in vitro*, as well as human neuronal precursors and astrocytes, leading to death by apoptosis or arrest of proliferation, respectively [16].

USUV infection has been shown to activate cellular stress response such as autophagy in the Vero cell line, which promotes its replication [17]. Infection of mammalian cells (human astrocytes and monocyte-derived dendritic cells, Vero and Hep-2 human cell lines) also activated innate immune responses and induced a high level of type 1 interferon (IFN) production [13, 15, 16]. In monocyte-derived DCs, USUV induced more type I IFN activity than both WNV lineages 1 and 2 [13]. Moreover, USUV replication was found to be more sensitive to types I and III IFNs than WNV replication [13]. These findings suggest that USUV is less efficient at counteracting IFN production than WNV and that USUV and WNV may interact differently with innate IFN antiviral defences.

In 1-week-old Swiss or NMRI mice infected intraperitoneally, USUV infection gives rise to clinical signs: disorientation, depression, paraplegia, paralysis and coma, and are associated with neuronal death in the brains of infected animals as well as demyelination of the spinal cord [18, 19]. In these studies, all of the suckling mice that survived to USUV infection were protected against a lethal challenge with a highly virulent WNV strain, suggestive of WNV clinical cross-protection afforded by USUV infection. However, USUV immunity did not reduce WNV replication upon subsequent WNV challenge. Unlike WNV, no mortality was recorded in adult mice (8 weeks old) infected with USUV at any of the doses tested, illustrating the limited pathogenicity of USUV in immunocompetent mice as compared to WNV [19, 20].

In contrast to immunocompetent adult mice, mice lacking the interferon *α*/*β* receptor (IFNAR-/-) were highly sensitive to USUV neuroinvasive infection, with death induced approximately 6 days after infection [21]. Moreover, high levels of USUV genomic RNA was detected in mouse brain samples. USUV neuroinvasive infections are also described in avian reservoir species, as well as in some human patients exposed to the virus (see below).

## Epidemiology

### Geographical distribution

**Fig. 1.**
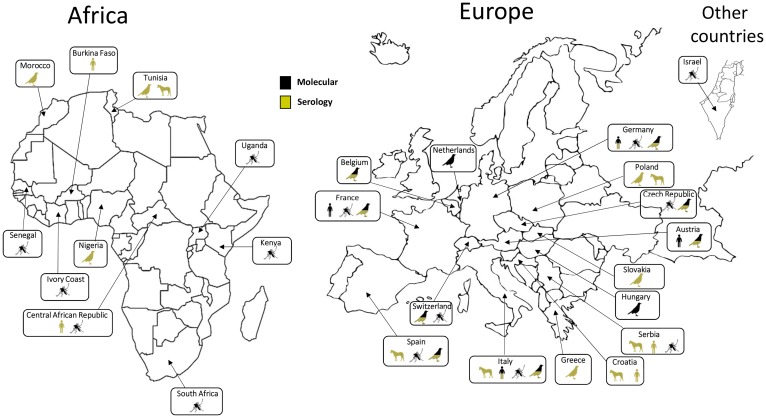
Worldwide USUV distribution. Concerned countries: Austria, Belgium, Burkina-Faso, Central African Republic, Croatia, Czech Republic, France, Germany, Greece, Hungary, Israel, Italy, Ivory Coast, Kenya, Morocco, Nigeria, The Netherlands, Poland, Senegal, Serbia, Slovakia, South Africa, Spain, Switzerland, Tunisia, Uganda. Symbols indicate in which species USUV has been detected (man, birds, mosquitoes or horses). Method of identification (molecular or serological) is indicated for each species.

Following its first identification in South Africa, USUV has been detected in other African countries: Central African Republic, Senegal, Ivory Coast, Nigeria, Uganda, Burkina Faso, Tunisia and Morocco [22–25]. This virus was also detected in Israel in *Culex* mosquitoes collected in 2014–2015 [26]. Phylogenetic analyses suggest that at least three USUV introductions have occurred in Europe along the migratory routes from Africa. The virus is thought to have been introduced in Spain on two occasions in the 1950s and then in the 1990s along an eastern Atlantic migratory route [10]. Furthermore, a unique introduction in central Europe appears to have occurred in the 1980s along a Black Sea/Mediterranean migratory route [10, 27]. Up to 2015, USUV infection had been reported from mosquitoes, birds or horses in 12 European countries (Germany, Austria, Belgium, Croatia, Spain, France, Greece, Hungary, Italy, the Czech Republic, Serbia and Switzerland) [28–32] (Fig. 1). During the summer of 2016, a major USUV epidemic affecting the avifauna was evidenced in Northern Europe, with extensive circulation in Belgium, Germany, France and, for the first time, in the Netherlands [8, 33, 34]. Furthermore, USUV infection has also been serologically identified in Slovakia and in Poland in equine and avian populations [35, 36]. In 2018, USUV spread rapidly in Western Europe, also associated with a large WNV epidemic that reached 1503 human cases, including 181 deaths in a dozen European countries [37, 38]. These data not only suggest a continuous geographical spread of the virus, but also the colonisation of new ecological niches. USUV endemicity in different European countries, as assessed by repeated transmission reports every summer and autumn, could be explained by residual enzootic transmission in affected areas but without detectable and significant clinical expression in bird populations. Moreover putative mechanisms of USUV persistence between two epizootic events involve USUV overwintering in infected mosquito females or in natural reservoir hosts or by virus vertical transmission of infected mosquito females to their offspring [39]. However, the mechanisms that allow the efficient overwintering and subsequent amplification of USUV in Europe have not been elucidated. The USUV strains identified in Europe display a broad genetic diversity, underscoring several introductions from Africa and the plasticity of the strains circulating in Europe. USUV frequently co-circulates with WNV in numerous European countries. WNV re-emerged in 2015 in Southeast France concomitantly with USUV, and enhanced dual reporting of WNV and USUV outbreaks in 2018 was observed in several European countries [40].

Given that USUV and WNV are genetically, antigenically and epidemiologically closely related, one question is whether such overlaps in transmission cycles can influence the spatiotemporal dynamics of the circulation of the two viruses in Europe and the associated risks for humans. Co-infections in humans would therefore be possible and probable. They could complicate diagnosis and symptomatology. In addition, since these two viruses are quite similar, ‘cross-immunity’ would be possible, which would make epidemiological models more complex. USUV, which appeared more than 20 years ago in Europe, has spread over the last years to many European countries with significant bird mortality in countries facing central European USUV strain circulation for the first time.

### Vertebrate hosts

USUV is maintained through an enzootic cycle between passerine birds mainly blackbirds (*Turdus merula*) or magpies (*Pica pica*) and Strigiformes, such as the Great Gray Owl (*Strix nebulosa*) as amplifying hosts and ornithophilic mosquitoes as vectors. USUV and WNV transmission cycles are therefore similar. USUV has been shown to infect 58 bird species derived from 13 orders and 26 families [41]. USUV can infect different European migratory bird species such as *Falco tinnunculus* (the common kestrel), *Sylvia curruca* (the lesser whitethroat) or *Ficedula hypoleucas* (the European pied flycatcher) [22] but also resident species, such as *P. pica* (the Eurasian magpie), *Passer domesticus* (the house sparrow) and *Turdus merula* (the common blackbird) [41] (Table 1). The virus was first identified in dead blackbirds in Austria in 2001 and in Italy in 1996 [42]. USUV was also isolated in captive owls found dead or moribund in zoological gardens in Austria (2001), in Switzerland (2006) and in France (2016–2018) [37, 43, 44]. Central nervous system disorders have been reported in USUV-infected birds. The most reported clinical signs are being prostration, disorientation, ataxia and weight loss. Hepatomegaly and splenomegaly are the main macroscopic lesions. Necrotic areas and inflammatory infiltrates composed of lymphoid and histiocytic cells have also been reported in the heart, liver, kidneys, spleen and brains of infected birds [45]. Glial nodules and neuronophagia have also been observed in the brain [45]. USUV can therefore be highly pathogenic in wild and captive birds, due to its wide tropism and virulence in a variety of tissues and organs. USUV circulation has consequently led to substantial avian death in different European countries although the consequences of USUV-associated mortality on the dynamics of avian populations have not been clearly investigated to date. The correlation between enhanced bird mortality and speed of virus turnover within the natural reservoir with the risk of USUV infection in incidental hosts such as humans needs to be evaluated, as for WNV [46].

**Table 1.** List of birds with USUV clinical infections

Order	Common name	Scientific name	Migration pattern		Countries with USUV RT-PCR positive animals	Ref
Passeriformes	Blackbird	*Turdus merula*	R,P		AT, CZ,FR,DE,HU,IT,NL,BE, CH	[32, 33, 44, 47–55]
	Common Starling	*Sturnus vulgaris*	R,P,S		DE, IT	[47, 49, 53, 55]
	Song Trush	*Turdus philomelos*	M		AT, DE, SP	[50, 55, 56]
	Canary	*Serinus canaria domestica*		Captive	DE	[49, 55]
	House sparrow	*Passer domesticus*	R		AT, DE, CH	[44, 48, 49, 53, 55]
	Blue (great) tit	*Parus caeruleus (major)*	R,P,M		AT, CH	[44, 48]
	European greenfinch	*Chloris chloris*	P		CH	[44]
	European robin	*Erithacus rubecula*	P		AT, CH	[44, 48]
	Bullfinch	*Pyrrhula pyrrhula*		Captive	BE	[57]
	Nuthatch	*Sitta europaea*	R		AT	[48]
	Eurasian Jay	*Garrulus glandarius*	R, P		HU,IT	[47, 53, 54]
	Magpie	*Pica pica*	R		IT	[52, 53]
	Barn Swallows	*Hirundo rustica*	M		AT	[42]
Strigiformes	Great Grey owl	*Strix nebulosa*		Captive	AT, DE, CH, FR, IT, NL	[33, 44, 48, 49, 55]
	Long-eared owl	*Asio otus*		Captive	DE,IT	[47, 55]
	Snowy owl	*Bubo scandiacus*		Captive	CH	[44]
	Tengmaml's owl	*Aegolius funereus*		Captive	CH, IT	[44]
	Hawk owl	*Surnia ulala*		Captive	CH, DE	[48, 55]
	Pygmy owl	*Glaucidium passerinum*		Captive	CH	[44]
Coraciiformes	Common Kingfisher	*Alcedo atthis*	R,P,M		DE	[49, 55]
	European Bee-eater	*Merops apiaster*	M		IT	[53]
Piciformes	Great spotted woodpecker	*Dendrocopos major*	R		BE	[14, 53]
	European Green Woodpecker	*Picus viridis*	R		DE, IT	[55]
Charadriiformes	Inca Tern	*Larosterna inca*		Captive	DE	[55]
	Yellow-legged gull	*Larus michahellis*	R		IT	[53]
Accipitriformes	Greater Spotted Eagle	*Aquila clanga*	M		IT	[53]
Caprimulgiformes	Nightjar	*Caprimulgus europaeus*	M		IT	[47]
Pelecaniformes	Grey Heron	*Ardea cinerea*	R, P		IT	[53]
Columbiformes	Collared Dove	*Streptopelia decaocto*	R		IT	[47, 53]
Galliformes	Red-legged Partridge	*Alectoris rufa*	R		IT	[47]

R, resident; P, partial; M, migratory; S, short distance.

Bird orders, common and scientific names and their behaviour (resident or migrating birds), as well as the countries having reported USUV positive RT-PCR animals are indicated.

Beyond birds, USUV has also been detected in mammals. USUV has been isolated from the brain of bats (*Pipistrellus)* found dead in southwest Germany, questioning the role of these animals in USUV amplification [58]. Other species can also be infected with USUV although the consequences of USUV exposure in these species have only been partially assessed. USUV-specific antibodies have been detected in the serum of horses in Italy, Serbia, Croatia, Poland and on the island of Mallorca in Spain [36, 59]. Virus neutralisation tests carried out on the sera of military horses and dogs in Morocco in 2012 also suggest exposure of these animals to USUV [24]. In 2014, another study reported the presence of anti-USUV antibodies in 10 equines in the southwest of Tunisia [23]. USUV-specific neutralizing antibodies have been detected in wild boars in Serbia [60]. Lastly, a retrospective serological survey, undertaken on the sera of 4693 wild ruminants has reported a prevalence of USUV-specific antibodies corresponding to 0.1–0.2% of the tested animals [61]. This study involved samples from Red deer (*Cervus elaphus*), Fallow deer (*Dama dama*), European mouflon (*Ovis aries musimon*) and Roe deer (*Capreolus capreolus*), collected between 2003 and 2014 in Spanish hunting parks. Serological tests (ELISA and serum neutralisation) have shown the circulation of USUV in hunting dogs in southern Italy (1.3% of the tested animals) [62]. More recently USUV has been isolated from rodent and shrew species in Senegal [63].

### Vectors

Several mosquito species are involved in WNV and USUV infection of the wild or captive avifauna [43, 64]. These mosquitoes are mainly ornithophilic species of the *Culex* genus. They are also responsible for virus transmission to susceptible mammals in particular to humans (WNV, USUV) and horses (WNV), which are viewed as incidental dead-end hosts, with short-lasting and low-level viraemia. USUV has been isolated from many species of mosquitoes throughout the African continent, primarily in countries where entomological surveillance programmes have been implemented, such as Senegal, Kenya and Uganda [22, 65] as well as more recently in southern and central Europe as in Italy or Austria [66]. The mosquito species in which USUV has been detected most often belong to the *Culex* genus like *Cx. modestus*, *Cx. neavei*, *Cx. perexiguus*, *Cx. perfuscus*, *Cx. pipiens*, *Cx. quinquefasciatus*, *Cx. univittatus* but also to other genera such as *Ae. albopictus*, *Ae. japonicus*, *Ae. minutus*, *Anopheles maculipennis*, *Culiseta annulata*, *Mansonia africana*, *Ma. aurites* (recently renamed *Coquilletidia aurites*), *Ochlerotatus caspius and Oc. detritus* (both formerly named *Ae. caspius* and *Ae. detritus*) (Tables 2 and 3). *Culex pipiens*, an ornithophilic species, but which can also feed on humans, is considered to be the main vector in Europe [47].

**Table 2.** Mosquito species found infected by USUV in the field and bridge vectors

Species (bridge vector)	Country (site)	Year	Reference
*Aedes albopictus* (S)	Israel (Haifa)	2015	[26]
	Italy (Emilia-Romagna)	2009, 2010, 2011, 2012	[47, 53, 67–69]
*Aedes japonicus*	Austria (Graz)	2018	[66]
*Aedes minutus* (N)	Senegal (Kedegou)	1998	CRORA (unpublished)
*Anopheles maculipennis* sl (S)	Italy (Emilia-Romagna)	2010, 2011	[53, 69]
*Culex antennatus* (S)	Senegal (Barkedji)	2012, 2013	[70]
*Culex modestus* (P)	Czech Republic (South-Moravia)	2013	[71]
	Italy (Emilia-Romagna)	2013	[72]
*Culex neavei* (S)	Senegal (Barkedji)	2003	CRORA (unpublished)
	Senegal (Barkedji)	2012, 2013	[70, 73]
	South Africa (Natal)	1959	[74]
*Culex perexiguus* (S)	Israel (Sdeh Eliahu, Midrach)	2015	[26]
	Spain (Guadalquivir Delta)	2009	[75]
*Culex perfuscus* (S)	Central African Republic	1969, 1980	Institut Pasteur de Bangui (unpublished)
	Senegal (Kedegou)	1974, 1998	CRORA (unpublished)
*Culex pipiens* (P)	Austria (Linz, Graz)	2018	[66]
	France (Camargue Delta)	2015	[31]
	Germany (Emsdetten)	2016	[30]
	Germany (Freiburg)	2014	[30]
	Germany (Leipzig)	2015	[76]
	Germany (Weinheim)	2010	[77]
	Israel (Kityat Ata)	2014	[26]
	Israel (Yeftachel)	2015	[26]
	Italy (Emilia-Romagna)	2009, 2010, 2011, 2012, 2013, 2014, 2015, 2016	[47, 53, 67–69, 78]
	Italy (Friuli-Venezia-Giulia)	2012	[69]
	Italy (Lombardy)	2009, 2011, 2013, 2014	[69]
	Italy (Lazio)	2018	[79]
	Italy (Liguria)	2014	[69]
	Italy (Marche)	2011	[69]
	Italy (Molise)	2011	[69]
	Italy (Piedmont)	2009, 2010, 2011, 2014	[69, 80, 81]
	Italy (Sardinia)	2011, 2013	[69]
	Italy (Tuscany)	2009, 2010	[52, 69]
	Italy (Veneto)	2009, 2010, 2011, 2012, 2013	[69, 82, 83]
	Serbia (Titel, Zrejanin)	2014	[29]
	Spain (Ebre Delta)	2006	[84]
	Switzerland (Ticino, Geneva)	2011, 2012	[85]
*Culex quinquefasciatus* (P)	Ivory Coast	2004	CRORA (unpublished)
	Kenya (Kisumu)	2007–2012	[65]
*Culex univittatus* (group) (S)	Senegal (Barkedji)	1993	CRORA (unpublished)
	Uganda (Jinja)	2012	[25]
*Culiseta annulata* (P)	Italy (Molise)	2011	[69]
*Mansonia africana* (S)	Central African Republic	1969, 1980	Institut Pasteur de Bangui (unpublished)
*Mansonia aurites*^a^ (S)	Uganda (Entebbe)	1962	[4]
*Ochlerotatus caspius*^b^ (S)	Italy (Emilia-Romagna)	2011, 2012, 2013	[53, 69, 72]
	Italy (Veneto)	2010	[69]
*Ochlerotatus detritus*^c^ (P)	Italy (Molise)	2011	[69]

P, potential; S, small probability; N, no probability.

Mosquito species and then countries are ordered alphabetically. Bridge vectors. P: refer to potential bridge vectors, i.e. mosquito species that readily bite birds and humans. S: refers to species with a lower probability of being bridge vectors and encompass opportunistic species that rarely bite both humans and birds, or have a low vector competence for WNV. N: refers to species that have very low or no probability of being a competent bridge vector. For Africa, USUV has been isolated only in countries in which entomological surveillance programmes have been undertaken particularly Senegal and Uganda, suggesting that its geographic distribution may be much wider than the reported detection.

a Recently renamed *Coquilletidia aurites*.

b Formerly named *Aedes caspius*.

c Formerly named *Aedes detritus*.

**Table 3.** Oral infection experiments. Infection, dissemination and transmission rates for mosquitoes 14 days after oral exposure to USUV

Species/(populations)	USUV Strain used	Blood meal titer (PFU/ml) (TCID50/ml)	Infection rate^a^	Dissemination rate^b^	Transmission rate^c^	Reference
*Culex neavei* (Barkedji, Senegal)	SAAR 1776	2 × 10^7^	33.3% (1/3)	0% (0/1)	–	[86]
		2 × 10^7^	22.2% (2/9)	0% (0/2)	–	
		2 × 10^7^	0% (0/1)	–	–	
		1.8 × 10^8^	**90.9%** (40/44)	**40.0%** (16/40)	**81.3%** (13/16)	
*Culex pipiens* (Brummen, The Netherlands)	USUV, Bologna '09	4 × 10^7^	**80%**	Not studied	**69%**	[87]
*Culex pipiens* (Mercer County, NJ, USA)	SAAR 1776	1 × 10^7.5^	**58.6%** (17/29)	**92.3%** (12/13)	**23.5%** (4/17)	[88]
*Culex quinquefasciatus* (Vero Beach, FL, USA)	SAAR 1776	1 × 10^6.95^	**70.0%** (21/30)	**35.7%** (5/14)	**19.0%** (4/21)	[88]
*Aedes albopictus* (Mercer County, NJ, USA)	SAAR 1776	1 × 10^5.95^	**0%** (0/27)	–	–	[88]
*Culex pipiens* form pipiens (Caldbeck, UK) *Culex pipiens* hybrid form (Brookwood, UK)	SAAR 1776	1 × 10^6^ (25°)	**5%** (1/20)	**5%** (1/20)	**100%** (1/1)	[89]
	SAAR 1776	1 × 10^6^ (25°)	**0%** (0/18)	–	–	
*Aedes albopictus* (Emilia-Romagna, Italy)	USUV1	0.66×10^7.5^	**0%** (0/20)	–	–	[78]
	USUV2	0.66×10^7.5^	**0%** (0/20)			
	USUV3	0.66×10^7.9^	**0%** (0/19)			

PFU, plaque-forming unit; TCID50, tissue culture infectious dose 50%.

After 14 days incubation at 27–28 °C and 80% relative humidity (except for Hernández-Triana *et al*., 2018 for which both UK lines of *Culex pipiens* were tested for their vector competence for the SAAR-1776 strain of USUV at 25 °C), fed mosquitoes were analysed for USUV infection of their bodies (infection), of their legs and wings (dissemination), and the presence of virus in the saliva (transmission).

a No. infected mosquito bodies/no. mosquitoes tested.

b No. mosquitoes with infected wings and legs/no. infected mosquitoes.

c Formerly named *Aedes detritus*

In addition, the vector competence of *Cx. pipiens*, *Cx. neavei* and *Cx. quinquefasciatus* for USUV has been demonstrated under laboratory conditions [86–88] (Tables 2 and 3). The vector competence of *Cx. pipiens* has been shown to be greater for USUV than for WNV, under conditions of elevated temperature (at 28 °C) [87]. In a recent study, two UK strains of *Cx. pipiens* challenged with an African strain of USUV showed a very low vector competence [89]. These contradictory results with previous experimental infections could be explained by the genetic variability of the USUV strains and the differences in susceptibility between different populations of the same mosquito species for the same virus [90]. The selective pressures associated with the laboratory colonisation process of mosquito populations can modify susceptibility to infection; moreover, experimental conditions, such as virus titres in the blood meal during oral infection and incubation temperature and length can also influence mosquito competence.

In contrast, North American and European populations of *Ae. albopictus* appear to be resistant to USUV infection even though this species has been repeatedly found infected in the Emilia-Romagna region, Northern Italy [78, 88, 91]. Isolation of infectious viruses or detection of viral RNA from *Ae. albopictus* may be a consequence of recent engorgement from viraemic avian species as some *Ae. albopictus* populations have been demonstrated to have opportunistic feeding behaviours and utilise avian species as a source of blood meals. Nevertheless, experimental infection studies of *Ae. albopictus* by USUV should be repeated, possibly using other vector populations, virus strains and dosages. Additional research should be carried out in the laboratory (vector competence) and in the field (vector capacity) to clarify its role in USUV (and also WNV) transmission and the associated risk for humans [78].

## Clinical manifestations in humans

The zoonotic potential associated with USUV infection was initially described in Africa. The first case of human infection by USUV was reported in the Central African Republic in the 1980s and a second case was diagnosed in Burkina Faso in 2004 [22]. For these two cases, mild clinical signs were reported: fever and skin rash. In Europe, the recent epizootics were also accompanied by descriptions of neuroinvasive infections in humans. In 2009 in Italy, two cases of meningoencephalitis associated with USUV infection were described in immunosuppressed patients [92, 93]. Shortly after this first description, three additional cases of USUV meningoencephalitis which occurred in 2008 and 2009 were retrospectively detected [94]. Again in Italy and during the same period, a retrospective study carried out recently in Emilia-Romagna region has allowed the documentation of eight other patients with encephalitis or meningoencephalitis, with USUV infection associated with other comorbidities in half of these cases, and two patients with asymptomatic infection [95]. Six other symptomatic cases were reported in Croatia in 2013 and in 2018 [96, 97]. These acute infections however do not reflect the full spectrum of human USUV infections, as the studies were carried out on cohorts exhibiting signs of neurological infections of varying severity. Our team recently described an acute USUV infection associated with a probable atypical presentation of *a frigore* facial paralysis in France [98]. The full clinical presentation of USUV infection needs thus to be better defined.

Recent studies performed on sera from blood donors have confirmed the existence of asymptomatic USUV infections [79]. This was the case in Germany for an asymptomatic blood donor who was found to be positive for USUV by PCR [99], and in Austria, for six donors who were positive for USUV [100]. In these two studies, primary screening of the blood donations for WNV, resulting positive in WNV screening tests allowed the identification of USUV infections by sequencing. Thus, out of the seven positive signals obtained by WNV RT-PCR in the Austrian study, six were identified as USUV after sequencing [100].

To date, in Europe, there have been 46 documented cases of acute USUV infection in humans, most of them were accidentally identified in donated blood samples. (Table 4). It is impossible to know whether these cases of infection represent the tip of the iceberg and whether the incidence of acute infections by USUV could, in fact, be more substantial. Reporting of USUV bird epizootics probably implies a higher exposure level of humans to the zoonotic risk. Indeed, the human cases detected in Italy coincided with USUV outbreaks, and interestingly some authors suggest that USUV exposure may be higher than for WNV [101]. The recent infections detected in blood donors in Germany and in Austria coincided with the most substantial epizootics that have been observed in recent times in central Europe, particularly in 2016 and 2018 [99, 100, 102]. Phylogenetic analysis of the viral strains detected in humans in these two countries identified at different period times strains of the Europe 2, Europe 3 and Africa 3 lineages corresponding to the same strains isolated in the populations of blackbirds and passerines from Germany and Austria. The strain derived from the Africa 2 lineage, implicated in the human case detected in the South of France [98], was also identified in mosquito populations of the *Cx. pipens* species captured nearby in the same region 1 year earlier [31]. Substantial circulation of USUV in avian reservoirs as well as in vectors appears to increase the probability of human infections.

**Table 4.** Chronological description of human cases worldwide

Acute infections (*n* = 49)							
Country	Year	Number	Sample	Clinic	Studied population	Diagnostic method	ref
CAR	1981	1	Blood	Eruptive fever	Clinical case	Culture	[22]
Burkina	2004	1	Blood	Fever and jaundice	Clinical case	Culture	[22]
Italy	2009	1	CSF	Meningoencephalitis	Clinical case	Panflavi RT-PCR + sequence	[92]
	2009	1	Blood	Encephalitis	Clinical case	Procleix-WNV + panflavi RT-PCR + sequence	[93]
	2008–9	3/44	CSF	Meningoencephalitis	Meningoencephalitis patients	Specific RT-PCR	[94]
	2008–11	8/306 + 2/609	CSF + blood	Meningoencephalitis /healthy	Meningoencephalitis patients (CSF) + various healthy and sick subjects (serum)	Specific RT-PCR + seroneutralisation	[95]
Croatia	2013	3/95	Blood	Meningoencephalitis	Meningoencephalitis patients	ELISA + seroneutralisation	[96]
	2018	3/178	Blood + Urine	Neuroinvasive disease	Neuroinvasive cohort	Specific RT-PCR + seroneutralisation	[97]
Germany	2016	1	Blood	Healthy	Blood donors (n ?)	Cobas WNV + sequence	[99]
France	2016	1/666	CSF	Idiopathic facial paralysis	Patients with infectious and/or neurological signs	RT-PCR panflavi + sequence	[98]
Austria	2017	6/12 047	Blood	Healthy	Blood donors	Cobas WNV + sequence	[100]
	2018	18/31 598	Blood	Healthy	Blood donors	Cobas WNV + specific RT-PCR + sequence	[102]

Seroprevalence (*n* = 98)						
Country	Year	Number	Prevalence (%)	Studied population	Diagnostic method	ref
Italy	2009	4/359	1.1	Blood donors	ELISA + seroneutralisation	[101]
	2008–11	40/609	6.5	Healthy patients and various sick subjects	Seroneutralisation	[95]
	2010–11	14/6000	0.23	Blood donors	ELISA + seroneutralisation	[103]
	2012	24/3069	0.78	Blood donors	ELISA + seroneutralisation	[104]
	2014–15	6/33	18.1	Healthy forestry worker	Seroneutralisation	[105]
	2014–15	2/200	1	Blood donors	Seroneutralisation	[103]
Serbia	2015	7/93	7.5	Healthy exposed subjects	ELISA + seroneutralisation	[106]
Germany	2012	1/4200	0.02	Blood donors	ELISA, IF + seroneutralisation	[107]

CAR, Central African Republic.

Seroprevalence studies seem to indicate non-negligible exposure of humans to USUV infection risk (Table 4). These studies carried out in Italy, in Germany and in Serbia reported USUV-antibody prevalences between 0.02% and 1.1% among healthy blood donors [101, 103–107]. Several of these studies show that USUV circulates more actively than WNV in Europe. Studies on the prevalence of USUV infection in mosquito vectors in Europe have revealed higher infection rates for USUV than for WNV. In Northern Italy (Emilia-Romagna), the Maximum Likelihood Estimates (MLE) value calculated as a regional seasonal average for *Cx. pipiens* was quite stable showing a continuous circulation at similar levels from 2009 to 2016 in the range 0.23–0.54 [78]. In contrast, WNV infection rates in the same mosquito species and in the same region were 2–5 times lower [53], with different strains circulating discontinuously and in different locations over the years [108]. This situation potentially reflects higher levels of circulation of USUV relative to WNV at least in some European regions like Northern Italy. Nonetheless, the data remain tenuous for accurately assessing USUV incidence in humans and the serological diagnostic tools available need to be improved to allow for large-scale screening.

## Diagnosis and surveillance of USUV infection

Diagnosing USUV infection in humans relies on several techniques: (i) the detection of viral RNA in blood and in cerebrospinal fluid (CSF), (ii) the isolation of the virus in cell culture and/or (iii) indirect assay detecting anti-USUV antibodies (IgM and G) in the serum and the CSF of patients.

To date, no commercial diagnostic test is available. USUV serological assays are based on ELISA tests or immunofluorescence tests that have been developed by reference laboratories, performed with viral antigens or virus isolates. These tests suffer from a lack of specificity. They need to be systematically confirmed by seroneutralisation assays to reduce the risk of serological cross-reactions described with infections by closely related flaviviruses, such as WNV. The kinetics of USUV antibody response in humans is not known and interpretations are usually drawn from data gained from WNV descriptions. Thus, in our recent experience, there was no detectable antibody response 3 days during an acute USUV infection, in contrast to the kinetics usually observed with other flaviviruses. Direct diagnosis of USUV infection can be obtained by isolating the virus in cell cultures and visualizing cytopathic effects. Numerous cells are permissive to the virus, and the most used are mosquito C6/36 or mammalian Vero cells [14].

The techniques used for the amplification of WNV RNA, from donated blood (such as the cobas^®^ WNV test (Roche Diagnostics, Germany)), also present a lack of specificity, allowing as well the detection of USUV genome [8, 100]. Numerous RT-PCR techniques have been described and some PCR methods have been developed to be specific for USUV sequences [94, 109] and others amplify USUV as several other flaviviruses by screening for a conserved region in the NS5 polymerase gene that is common to these viruses [110–112]; virus typing can then be performed secondarily by sequencing or hybridisation [111]. This ‘pan-flavivirus’ approach is certainly more cumbersome but it provides a double advantage. First, it offers a wider range of detection, which may be useful for the integrated surveillance of different arboviruses with very similar epidemiology, such as USUV and WNV. Second, the sequencing step, necessary for the identification of the viral aetiology, also allows for a phylogenetic analysis of the strains. Parts of the *NS5* gene that are targeted by pan-flavivirus RT-PCR have proven to be sufficiently selective for the characterisation of viral lineages [8, 10]. Since 2010, Mediterranean regions as Italy and southern France have been monitored for the risk of WNV. This monitoring now includes a veterinary component and a human component with awareness among clinicians of the presumptions for aseptic meningitis. The risk of the emergence of USUV, which shares many genetic, antigenic and epidemiological features with WNV, should lead to the inclusion of USUV in monitoring programmes. Clearly, there is a need to organise standard surveillance measures and early warning systems to detect WNV and USUV activity, and to assess the risk for public health, both at the national and European level. The information gathered through these surveillance programmes could be used to develop actions to prevent virus transmission, such as vector prevention and control, information campaigns to improve personal protection as well as screening tests for blood donations, tissue and organs. The inclusion of USUV together with WNV in surveillance plans is of primary importance and has been implemented mainly in Italy. The lack of specificity of USUV/WNV diagnostic tools, whether serological or molecular, could be an advantage in this situation, provided that there is a full characterisation of the positive cases by confirmatory serological assays (virus neutralisation tests) and by sequencing or by virus-specific RT-PCR.

## Conclusion

Responsible for recurrent epizootics since 1996 in the European avifauna, USUV is now recognised as being responsible for potentially severe neurological affections in humans. Its recent spread to a large number of European countries and co-circulation of different genetic strains deserve increased awareness and characterisation. Furthermore, USUV has been shown to co-circulate with WNV in different areas raising epidemiological and diagnostic issues. Serological cross-reactions can hamper rapid identification of circulating viruses in the absence of material allowing for direct diagnosis and can offer partial cross-protection against the other flaviviruses, possibly influencing its amplification and transmission patterns. As for any emerging arbovirus, a multidisciplinary approach involving virologists, clinicians, ornithologists, entomologists as well as closer intersectoral collaborations between operations (health, agriculture, environment) and stakeholders (involving environment, veterinary and human sectors) following the One Health approach should be established. This would help bridging the data gaps in USUV epidemiology and identifying the main risk factors, with the aim of implementing appropriate monitoring and prevention methods.
